# Effects of non-extensible lumbar belts on static and dynamic postural stability

**DOI:** 10.1186/s12891-023-06476-w

**Published:** 2023-05-08

**Authors:** Jingyuan Bai, Anke Hua, Dongkai Weng, Nan Wang, Jian Wang

**Affiliations:** 1 Department of Sports Science, College of Education, Zhejiang University, Hangzhou, 310058 China; 2 Hangzhou Weizhen Health Technology Co., Ltd., 310058 Hangzhou, China; 3 Center for Psychological Sciences, Zhejiang University, Hangzhou, 310058 China

**Keywords:** Non-extensible lumbar belts, Intra-abdominal pressure, Postural stability

## Abstract

**Background:**

Previous studies have found that increased intra-abdominal pressure helps to reduce spinal loading and improve spine stability. Non-extensible lumbar belts (NEBs) could elevate intra-abdominal pressure and augment spinal stability. NEBs have been used in the healthcare field to help reduce pain and improve spine function for people with low back pain. However, the effect of NEBs on static and dynamic postural stability is not clear.

**Methods:**

This study aimed to investigate whether NEBs affect static and dynamic postural stability. Twenty-eight healthy male subjects were recruited to finish four static postural stability tasks and two dynamic postural stability tests. Center of pressure (COP) values during 30 s of quiet standing, dynamic postural stability index (DPSI) and Y balance test (YBT) score with and without NEBs were analyzed.

**Results:**

NEBs had no significant effect in all COP variables in the static postural tasks. The results of a repeated measure two-way ANOVA indicated the NEBs significantly improved the dynamic postural stability in YBT score and DPSI (F _(1,27)_ = 5.506, *p* = .027, ${{\eta }_{p}}^{2}=.169$ and F _(1,27)_ = 83.94, *p* = .000, ${{\eta }_{p}}^{2}=.757$ respectively).

**Conclusions:**

The study results indicate that non-extensible belts improve dynamic stability in healthy male participants, with potential implications for rehabilitation and performance enhancement programs.

## Background

Lumbar belts (LBs) have been applied to protect the lumbar and relieve low back pain (LBP) [1]. People with LBP exhibited impaired postural stability during standing and increased trunk muscle activity during dynamic activities [2–4]. LBs are regarded to reduce the lumbar range of motion and enhance lumbar stiffness during maximal trunk flexion–extension and manual material handling tasks [5]. Although there is a small decrease in muscle activation with LBs [1, 6], they do not decondition the muscle, decrease spinal function and induce muscle fatigue wearing for a certain period of time [7, 8]. LBs reduce pain and decrease functional impairment for people with LBP [1, 6, 9].

The possible mechanism of LBs for relieving LBP is to improve spine stability and reduce spinal loading by increasing intra-abdominal pressure (IAP) [9, 10]. Studies show that increased IAP can promote co-contraction of spinal flexor and extensor muscles to increase trunk stiffness [10, 11]. Besides, IAP generates a longitudinal moment acting on the pelvis and diaphragm, which helps reduce intervertebral pressure and enhance spine stability [12]. Furthermore, transversus abdominis (TrA), in which bundles run horizontally, can transfer tension from the abdominal muscles to the lumbar spine, thereby regulating the segmental motion of the lumbar spine [13]. Biomechanical models have shown that TrA successfully compensates for other abdominal muscle co-activities [14]. LBs increase IAP by tightening the abdominal cavity like TrA to stabilize the spine. Also, it was reported that an adequate IAP increase leads to an enhancement of hip extension maximum voluntary contraction torque [15], which may help to improve stability. Above all, LBs seem to be simple and effective in augmenting IAP and improving spine stability.

Static and dynamic postural stability (PS) are essential for controlling the center of mass and maintaining equilibrium [16]. Typically, static PS is assessed by collecting the center of pressure (COP) trajectories during quiet standing with different visual or somatosensory conditions [17]. In contrast, dynamic PS reflects the ability to coordinate locomotor and maintain PS in sports and daily life [16, 18]. Previous studies have shown that dynamic PS tasks, such as landing and stabilizing after a jump, involve additional components of the neuromuscular systems compared to static posture tasks [19, 20]. One of the most commonly used clinical assessments for dynamic postural PS is the Y balance test (YBT), which has high reliability and validity [21]. However, YBT cannot capture the PS variability during dynamic posture transition [22]. To address this limitation, the dynamic postural stability index (DPSI) has been developed to measure stability during sudden transitions from a dynamic to a static state [23]. Previous work has shown that DPSI is an accurate and sensitive method for measuring the variability in ground reaction forces on landing [24–26]. Combining YBT and DPSI provides a comprehensive assessment of dynamic PS.

Several studies have revealed that LBs can improve PS by enhancing spine stability and supporting the lower back [1, 8, 27]. For instance, one study reported that the postural sway of people with LBP showed a 51% reduction during standing wearing a lumbar support [27]. In terms of the immediate effects of LBs on postural control, another study found that wearing LBs for 4 weeks improved static PS in anteroposterior and mediolateral directions among people with LBP [28]. Additionally, a systematic review revealed that external lumbar supports had positive effects on postural control in conditions where somatosensory interference was presented, such as LBP or standing on an unstable surface [29]. However, some studies did not reach the same conclusion. Some studies showed that LBs reduced posture sway in patients with LBP only when completing challenging postural tasks (i.e., single leg landing with closed eyes), while others indicated that lumbar supports mainly corrected posture mechanics and failed to affect postural control system [30–32]. These inconsistent findings highlight the need for more evidence regarding the effect of LBs on static PS. Despite the existing studies on LBs on static PS, the evaluation of dynamic PS changes is of great significance. To our knowledge, no study has yet examined the effect of LBs on dynamic PS by YBT or DPSI. Thus, in this study, we aim to use DPSI combined with YBT to investigate the impact of LBs on dynamic PS.

The effect of LBs for improving stability is closely related to the material and design of the LBs [9, 32]. Extensible and non-extensible belts (NEBs) are two common types of LBs. Extensible belts are elastic made of neoprene and lycra, while NEBs are made of polyester and nylon with fixed length [32, 33]. Besides, NEBs have a tensioning system with rigid anchors in the back and adjustable straps on both sides, which can increase intra-abdominal pressure [1]. While both types of LBs have been reported to enhance spinal stability in healthy adults, the materials and design of LBs may result in different outcomes in terms of their effects on spine stability [1, 33]. Several studies have found that NEBs increase trunk stiffness more significantly than extensible belts [7, 32, 34]. Moreover, a study that compared the effects of both types of belts on trunk stiffness with a pressure of 70 mmHg found that NEBs were more effective in restricting trunk motion following a perturbation [34]. Furthermore, wearing a NEB for two weeks significantly increased trunk extensor endurance for healthy participants who performed a modified Sorensen test, while wearing extensible belts for 21 days decreased trunk extensor endurance [7, 35]. Additionally, NEBs were found to lead to greater improvement in daily living function for LBP patients than extensible belts [32]. Therefore, it can be preliminarily considered that the NEB is a better lumbar belt option for enhancing IAP and spine stability.

Above all, the aim of this study is to investigate the effect of NEBs on static and dynamic PS in different static and dynamic postural tasks. As NEBs have been demonstrated to augment spine stability by inducing higher IAP, we hypothesized that NEBs contribute to improving static and dynamic PS in healthy subjects.

## Methods

### Participants

This study recruited 28 male subjects (age = 22 ± 2.07 years, height = 175.79 ± 5.82 cm, weight = 65.9 ± 7.84 kg). The dominant leg of all subjects was the right leg. The dominant leg was determined through a test to hit a soccer ball. The exclusion criteria were injuries of the lower extremity in the past six months and lower extremity surgery in the past two years and any musculoskeletal condition that might interfere with postural stability, such as lower extremity soreness or a neurological disorder. The sample size was calculated using G*Power software (Version 3.1 for Mac). The results indicated that a total of 24 participants were necessary with a significant level of 0.05, an effect size of 0.25 and a statistical power of 0.8 [36, 37].

### Experimental procedures

**Fig. 1:**
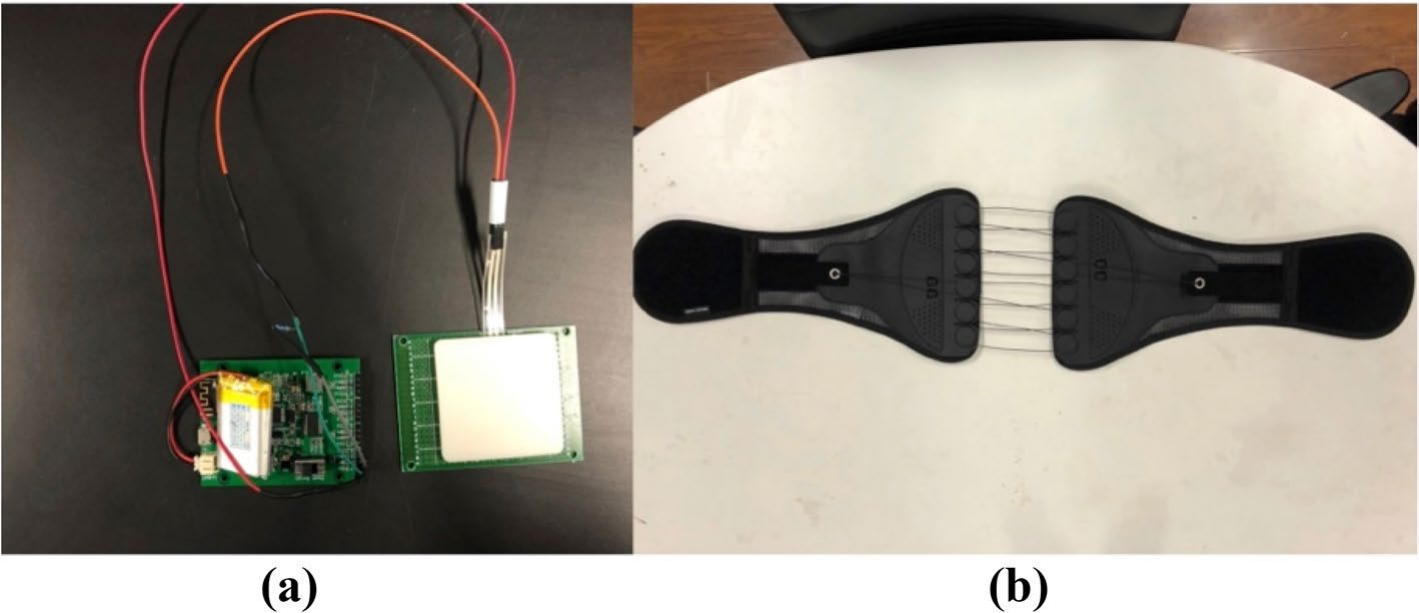
Diagram of (**a**) a pressure sensor; (**b**) a non-extensible lumbar belt

The subjects completed the static and dynamic PS tests with and without the NEB, respectively, of which the static PS test was standing still for 30 s under different conditions [38]. Dynamic PS tests included YBT and jump landing. The test order was pseudo-randomized and counterbalanced. Half of the subjects completed the tests with NEB first, and the other half completed the tests without NEB first. The time interval between trials was 30 s. The NEB applied pressure was set to 70 mmHg measured by a force sensor inserted between the above anterior superior iliac crest and the NEB (applied pressure = 77.76 ± 11.18 mmHg) [34] (Fig. 1).

### Static postural stability test

Static PS were tested using four paradigms: 1) Standing with eyes open on firm ground (EO); 2) Standing with eyes closed on firm ground (EC); 3) Standing with eyes open with a foam mat (EOMAT); 4) Standing with eyes closed with a foam mat (ECMAT) [39, 40]. The first task was chosen because it reflects the posture in natural state, and the latter ones were picked to disturb the visual and/or somatosensory systems. The foam mat used in this study was the Airex Balance Pad (Airex AG, Sins, Switzerland).

Subjects were requested to stand upright on a force platform (OR-6, 1000 Hz, AMTI, USA) with their arms at their sides, barefoot with feet together, remaining as stable as possible, and looking straight ahead at the black cross marked 1.5 m away on the wall.

### Dynamic postural stability test

Two dynamic PS tests were conducted, including YBT and jump landing. For YBT, subjects were asked to put their hands on their hips and place the most distal end of the longest toe of the stance leg barefoot at the red line on the platform on the test kit while the other leg gently pushed the baffle to the limit in anterior, posteromedial, and posterolateral directions [22]. In the whole process, the subjects need to maintain balance. If there were phenomena such as the swing leg falling and the stance leg moving off the platform, the attempts would not count. The assessment was conducted on both the non-dominant (N-Dominant) and dominant (Dom) legs. To obtain accurate measurements, three consecutive attempts were performed in each direction with both legs, and the maximum value was selected for data analysis [41]. All subjects were familiarized with the test after completing 4 to 6 practices trials.

For jump landing, participants were required to take off on both feet from a specific distance and jump over a hurdle and the dominant leg landing on the force platform and standing with hands-on-hips for 10 s, including anterior–posterior (AP) jump and medial–lateral (ML) jump. The force platform is flush with the surrounding ground. The jump distance equals 40% and 33% of the subjects' height in the AP and ML jump, respectively. The height of hurdles in the AP and ML jump is 30 cm and 15 cm, respectively [42]. If the non-stance leg of the subject touched the stance leg or force platform and the stance leg was moving, trials were discarded and retested. Two successful trials were required in each direction, and the mean value was used for analysis [43].

### Data processing

All data from the force platform were processed offline using customized MATLAB programs (R2021a, MathWorks, USA) and filtered using low-pass Butterworth filters with a cut-off frequency of 20 Hz. COP trajectories were calculated by Eq. (1) and Eq. (2), in which d (0.038 m) represents the height of the force platform itself [44]. Considering the influence of oral instructions by the investigator at the start and the end of the static PS test on COP trajectory, the first and the last 5 s of raw data were discarded. COP sway area (EA) was calculated with a 95% confidence ellipse area. COP path length (PL), COP velocity in the mediolateral (ML velocity) and anteroposterior (AP velocity) directions were also calculated. DPSI values of 3 s were calculated for the filtered GRF data according to Eq. (3) [24]. Data were cropped from the time of initial contact with the force platform, defined by a threshold of greater than 5.0% of body weight [45]. YBT data was calculated for the composite score using Eq. (4) [41].

1 $${COP}_{ML}=-\frac{{M}_{Y}+d\times {F}_{X}}{{F}_{Z}}$$

2 $${COP}_{AP}=\frac{{M}_{X}-d\times {F}_{Y}}{{F}_{Z}}$$

3 $$DPSI=\sqrt{\frac{\sum {(0-GRFx)}^{2}+\sum {(0-GRFy)}^{2}+\sum {(Body\,Weight-GRFz)}^{2}}{number\,of\,data\,points}}\div Body\,Weight$$

4 $$Composite\,Score=\frac{(Anterior+Posteromedial+Posterolateral)}{3\times Right\,Limb\,Length}\times 100\%$$

### Statistical analysis

SPSS software (version 20.0, IBM. USA) analyzed the processed data statistically. Data distribution was examined for normality using Shapiro–Wilk test (< 50 samples). T-tests and non-parametric alternatives Wilcoxon signed-rank test were used to analyze differences between without NEB and NEB conditions resulting from different static postural stability tasks. A repeated-measure two-way analysis of variance (ANOVA) design was used to assess the effect of NEB on YBT composite score (NEB × stance leg), and DPSI (NEB × jump direction). Bonferroni was used for post hoc. The statistical significance level was set as *p* < 0.05.

## Results

Twenty-eight male subjects finished four static PS tasks and two types of dynamic PS tasks. The data of static PS tasks were shown in Table 1. The static PS parameters related to the COP sway, i.e., EA and PL did not differ for without NEB conditions or NEB conditions either in eyes open nor eyes closed condition. Similarly, the stability parameters related to the COP velocity did not differ for without NEB conditions or NEB conditions.

**Table 1:** Results of 28 subjects in four static postural stability tasks

Parameter	Type	EO		EC		EOMAT		ECMAT	
		Without NEB	NEB	Without NEB	NEB	Without NEB	NEB	Without NEB	NEB
EA	Mean ± SD	303.8 ± 319.6 (107.9–420.4)	314.6 ± 296.3 (112.5–435.9)	405.3 ± 351.3 (206–554.3)	443.7 ± 209.5 (272.6–559)	970.5 ± 351.4 (711.9–1106.4)	758.7 ± 599.7 (362.3–1095.6)	2216.7 ± 1711.4 (1075.7–2786.9)	2106.2 ± 1631.8 (1274.2–2665.6)
	Range								
PL	Mean ± SD	302.7 ± 78.2 (250.2–344.3)	305.6 ± 55.5 (264.9–344.7)	417.2 ± 92.1 (350–470)	411.0 ± 90.4 (363.8–469.8)	543.3 ± 108.7 (459.7–603.8)	517.3 ± 126.8 (403.8–586.2)	1123.6 ± 343.9 (897.3–1341.1)	1136.6 ± 407.4 (916.1–1326.5)
	Range								
ML velocity	Mean ± SD	1.22 ± 0.35 (0.95–1.49)	1.23 ± 0.29 (1.00–1.35)	1.65 ± 0.44 (1.43–1.86)	1.61 ± 0.44 (1.29–1.91)	2.12 ± 0.43 (1.81–2.35)	2.13 ± 0.61 (1.70–2.51)	4.56 ± 1.30 (3.66–5.19)	4.65 ± 1.49 (3.69–5.05)
	Range								
AP velocity	Mean ± SD	1.32 ± 0.35 (1.08–1.57)	1.31 ± 0.24 (1.12–1.40)	1.82 ± 0.43 (1.54–2.03)	1.81 ± 0.45 (1.57–2.21)	2.46 ± 0.54 (2.01–2.94)	2.26 ± 0.53 (1.81–2.68)	4.89 ± 1.81 (3.89–5.52)	4.86 ± 2.08 (3.63–5.74)
	Range								

The values of all parameters are presented as mean ± standard deviation followed by 1st quartile and 3rd quartile in brackets

*EO* Eyes open on firm ground, *EC* Eyes closed on firm ground, *EOMAT* Eyes open with a foam mat, *ECMAT* Eyes closed with a foam mat, *EA* Elliptical area, *PL* Path length of COP trajectory, *ML* Mediolateral, *AP* Anteroposterior

**Fig. 2:**
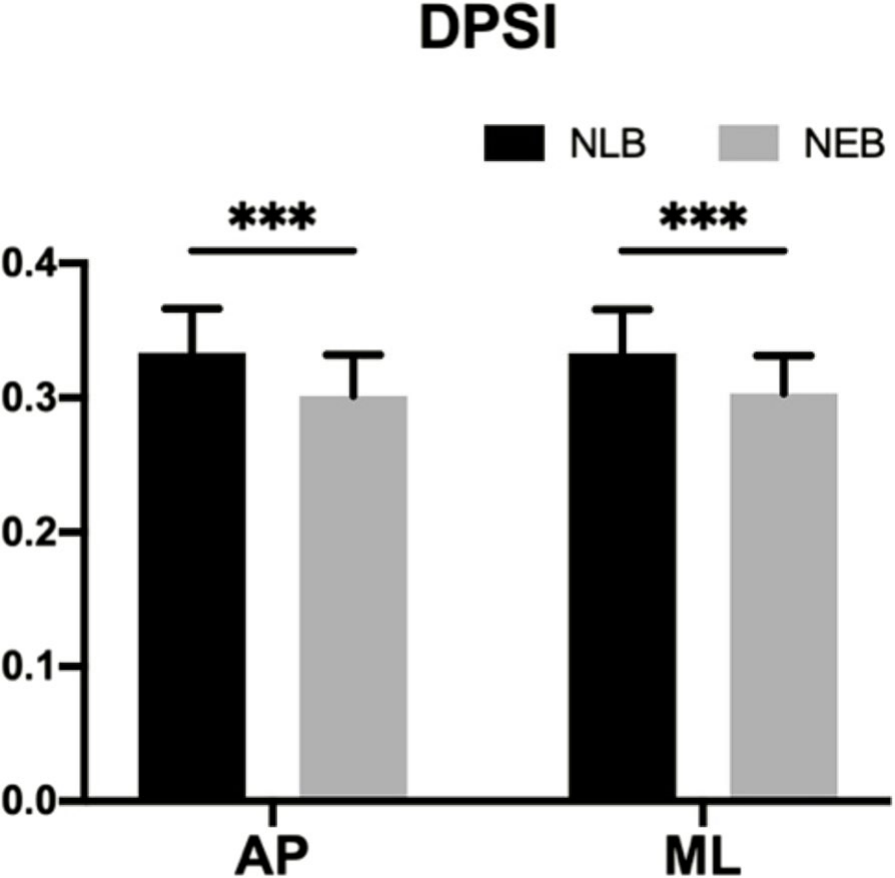
Results of statistical analysis of DPSI. NEB: non-extensible lumbar belt; NLB: no lumbar belt; AP: anterior–posterior jump; ML: medial–lateral jump; *** represents *p* < 0.001

The analysis results of DPSI are shown in Fig. 2. The main effect of NEB was significant (F _(1,27)_ = 83.94, *p* = 0.000, ${{\eta }_{p}}^{2}=.757$), but the main effect of the jump direction was not significant (F _(1,27)_ = 83.94, *p* = 0.850, ${{\eta }_{p}}^{2}=.001$), and the interaction effect of NEB and jump direction was not significant (F _(1,27)_ = 83.94, *p* = 0.783, ${{\eta }_{p}}^{2}=.077$).

**Fig. 3:**
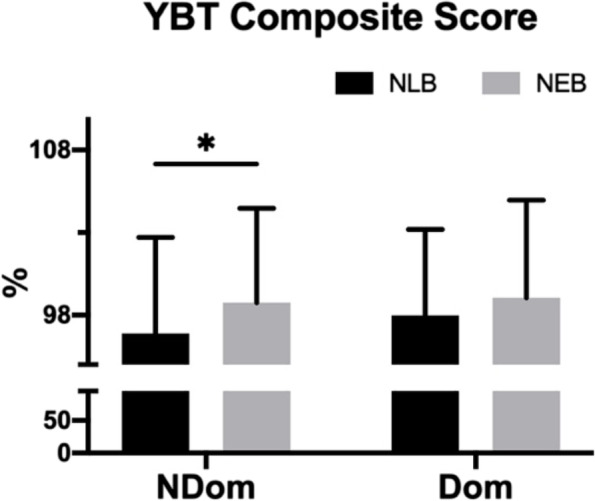
Results of statistical analysis of YBT composite score. NEB: non-extensible lumbar belt; NLB: no lumbar belt; NDom: non-dominant leg; Dom: dominant leg; * represents *p* < 0.05

The analysis results of YBT are shown in Fig. 3. The composite score showed a significant main effect of NEB (F _(1,27)_ = 5.506, *p* = 0.027, ${{\eta }_{p}}^{2}=.169$), and the main effect of the stance leg was not significant (F _(1,27)_ = 1.309, *p* = 0.263, ${{\eta }_{p}}^{2}=.046$), and the interaction effect of NEB and the stance leg was not significant (F _(1,27)_ = 1.867, *p* = 0.183, ${{\eta }_{p}}^{2}=.065$).

## Discussion

This study aimed to determine the effect of NEBs on static and dynamic PS in healthy participants. The main findings of this study were as follows: 1) the NEBs had no significant effect on the static PS in healthy male participants; 2). the NEBs were beneficial for healthy male subjects to improving dynamic PS.

No significant difference of COP values was revealed between NEB wearing and not wearing tasks, including in visual deprivation and somatosensory interference conditions. Previous studies about the effect of lumbar belts and orthosis rarely measured the COP of postural stability [27, 46]. Several relevant studies involving COP values showed that wearing orthosis contributed to reducing the postural sway [31, 47]. One study reported no significant changes in COP values (i.e., AP velocity and ML velocity) of the NEB group compared to the control group under open or closed eyes conditions, which is consistent with our findings [31]. Another study found that a lumbar orthosis reduced COP mean displacement by 51% for LBP patients during quiet standing with eyes closed [27]. However, the authors suggested that the degree of displacement was insufficient to explain the effects of the lumbar orthosis. Additionally, another study found that the lumbar orthoses improved static stability when standing on a foam mat [47]. A systematic review also showed the positive effects of LBs on static PS were observed in the case of somatosensory feedback impaired (i.e. LBP) or deprivation (i.e. standing on an unstable surface with eyes closed) [29]. In line with the results above, a study concluded that the effect of LBs is associated with the challenging of the postural tasks and the type of subjects [30]. In our study, quiet standing was a simple task for healthy participants, which made the NEB not show the effect of enhancing static PS. Furthermore, as previously described, the somatosensory acuity of the spine affects trunk muscle control and altered postural control [48, 49]. It was noted that the somatosensory function in the low back region of patients with LBP is compromised [50]. LBs were thought to could partly provide somatosensory information for the lumbar of LBP [51]. However, another study presented that LBs had no somatosensory benefits to healthy subjects [52], which might explain why the NEB did not improve static PS in our study. Future research should examine the effect of the NEB on elderly and LBP patients with balance disorders and somatosensory impairment.

In addition to static PS testing, more studies about postural control have begun to pay attention to dynamic PS. Nevertheless, to the best of our understanding, there hasn't been any prior research assessing the impact of the NEB on dynamic PS. Our study initially discovered that the utilization of NEB led to an improvement in dynamic PS among healthy male participants. The dynamic PS was assessed through two standardized tests that measured balance and stability, including YBT and DPSI. The results showed a statistically significant improvement in dynamic PS among participants who wore the NEB compared to those who did not. On average, the improvement of DPSI was 9.3% and 1.5% of YBT score higher in the NEB condition. These results suggest that utilizing NEBs may be a slightly effective intervention for improving dynamic PS in healthy males.

Our investigation found that the utilization of NEBs led to an average elevation of IAP to 70 mmHg. Previous research has demonstrated that IAP impacts body movement, which aligns with the decrease in DPSI observed in our study [53]. Additionally, a 30 mmHg increase in IAP has been shown to improve spinal stability by 25% [53], and targeted trunk muscle strengthening exercises have also been demonstrated to enhance spinal stability and postural control in individuals with LBP [54]. Similarly, heightened IAP has been shown to enhancing spinal stability [55]. These findings support our conclusion that the use of NEBs improve dynamic postural stability by enhancing spinal stability. Furthermore, one study reported a significant improvement in walking efficacy for LBP patients without causing erector spinae muscle fatigue after walking a NEB for a month, suggesting that NEBs may contribute to enhancing dynamic performance [8]. However, the effect of NEBs on dynamic postural stability is not yet fully understood and requires further investigation. Future research should aim to explore the long-term effects of NEBs on dynamic postural stability and the underlying mechanisms.

The Jump landing test presents a reliable and challenging evaluation of dynamic PS [45], which was initially designed to assess the dynamic balance of athletes. DPSI has been found to reflect not only the adjustment phase of the center of mass during impact, but also the kinetic energy absorption during the landing process [56], making it a comprehensive indicator of joint energy absorption and PS during jumping movements. Previous studies, as well as our own, have consistently demonstrated that there is no significant difference in DPSI between AP and ML jumps. This is likely due to the fact that DPSI primarily reflects the body's ability to absorb kinetic energy from the vertical peak force and is not strongly influenced by the direction of the jump [26, 57]. Therefore, while our findings and those of previous studies suggest that there may be no significant difference in DPSI between AP and ML jumps, further research is needed to confirm and expand upon these results. Addtionally, the correlation between spinal stability and dynamic PS has yet to be thoroughly investigated, despite studies indicating that ankle and knee flexion and extension strength predict dynamic PS [25]. Our findings suggest that improving spinal stability through the use of NEBs enhance dynamic postural stability, though further research is needed to fully understand the underlying mechanism of this relationship.

The improvement in dynamic PS with the use of NEBs has potential clinical applications, especially for individuals who are prone to falls or have difficulty with balance, such as athletes with lower extremity injuries, older athletes and patients with balance disorders. Besides, the results of our study suggest that the NEB may be a useful tool for enhancing dynamic PS. It can be applied in several areas of health, including sports rehabilitation, physical therapy and so on, to enhance the effectiveness of treatment and promoting positive outcomes for individuals.

This study had two limitations. First, the participants were only healthy male individuals and not include females or other special populations. Therefore, the results may not be generalizable to other groups. Secondly, the EMG data of muscles were not collected in static and dynamic standing posture tests, therefore this study failed to explore the physiological and neural control mechanism of NEB on static and dynamic PS. Future research should include both genders to increase the generalizability of the findings, as well as investigate the effectiveness of NEBs in populations with LBP or musculoskeletal injuries. These results suggest that NEBs may be a useful component of rehabilitation programs for improving balance instability.

## Conclusions

The results of our study showed that using non-extensible belts improved dynamic postural stability in healthy male participants, while static stability remained unchanged. These findings have implications for understanding the impact of non-extensible belts on postural stability and for the design and implementation of effective rehabilitation and performance enhancement programs. Further research is necessary to confirm these findings in other populations, including those with LBP, and to gain a better understanding of the mechanisms behind the improvement in dynamic stability.

## Data Availability

The dataset supporting the conclusions of this article is available from the corresponding author Jian Wang on reasonable request.

## Abbreviations

AP: Anterior-posterior
COP: Center of pressure
Dom: Dominant leg
DPSI: Dynamic postural stability index
EA: Elliptical area
EC: Eyes closed on firm ground
EO: Eyes open on firm ground
ECMAT: Eyes closed with a foam mat
EOMAT: Eyes open with a foam mat
IAP: Intra-abdominal pressure
LBP: Low back pain
LBs: Lumbar belts
NEBs: Non-extensible lumbar belts
NDom: Non-Dominant leg
ML: Medial-lateral
PL: Path length
PS: Postural stability
TrA: Transversus abdominis
YBT: Y balance test
